# Editorial: Reviews in pharmacology of anti-cancer drugs: 2023

**DOI:** 10.3389/fphar.2025.1667260

**Published:** 2025-10-02

**Authors:** Stefania Nobili, Donato Cosco

**Affiliations:** ^1^ Department of Neuroscience, Psychology, Drug Research and Child Health - NEUROFARBA - Section of Pharmacology and Toxicology, University of Florence, Florence, Italy; ^2^ Department of Health Sciences, University “Magna Græcia” of Catanzaro, Catanzaro, Italy

**Keywords:** cancer, drug delivery systems, antitumor compounds, anticancer therapies, drug targeting

Oncological research represents a cornerstone in the global effort to reduce the burden of cancers that are characterized by marked biological complexity and heterogeneity (Ng and Shaffer, 2023; Imodoye et al., 2024). A multifaceted strategy is required to address the aforementioned crictisms, drawing on approaches of molecular and cell biology, genomics, immunology, pharmacology, drug delivery and public health (Liu et al., 2024; Ammar et al., 2025). Each of them provides distinct and complementary insights, allowing a more holistic understanding of tumor development, progression, and response to therapies. In addition, the integration of various methodological approaches—ranging from *in vitro* and *in vivo* studies to clinical trials and epidemiological investigations—enhances the robustness and translational potential of research findings. The interdisciplinary paradigm has proven essential for fostering innovation and for the development of innovative diagnostic and therapeutic therapies in oncology (Mäurer et al., 2023; Grillone et al., 2024).

The contributions included in this Research Topic help shed light on some of these critical aspects, offering valuable perspectives on the various experimental and clinical approaches employed in cancer treatment.

Jiang et al. described the efficacy and safety of various treatments for patients with unresectable colorectal liver metastases (CRLM), with a specific focus on the comparison between first-line and maintenance therapies. Their network meta-analysis included 56 randomized controlled trials (RCTs) encompassing a total of 21,323 patients. Only phase II or III RCTs comparing two or more treatment strategies were included. The analysis revealed that, for first-line therapy, hepatic local treatment or targeted therapy combined with chemotherapy were among the most effective options. In particular, resection or ablation and the use of a single chemotherapeutic agent were associated with the best overall survival, while drug combination, such as atezolizumab + bevacizumab + fluorouracil-based chemotherapy, resulted in the longest progression-free survival. Monotherapy and local treatment were associated with fewer severe side effects, whereas combination therapies were associated with increased toxicity. The described findings provide valuable evidence on the efficacy abd safety of therapeutic strategies employed for the treatment of unresectable CRLM.

The treatment of hepatocellular carcinoma (HCC) represents a major global health challenge ranking as the sixth most common cancer worldwide. While surgery is still considered the first-line approach for the management of the disease, pharmacological therapies play an important role, especially in advanced stages of the disease. Chen et al. reviewed the current landscape of globally approved pharmacological treatments for HCC and highlighted the emerging trends from recent clinical trials, providing a perspective on future therapeutic innovations. In detail, treatment regimens based on single agents–including molecular targeted therapies and immune checkpoint inhibitors used alone or in combination - play a pivotal role in the management of HCC. However, the identification of optimal pharmacological treatments remains an unmet clinical need, and the development of more effective therapeutic agents is required.

Leptomeningeal disease (LMD) is a rare but severe tumor-related condition affecting the central nervous system, characterized by the dissemination of malignant cells within the subarachnoid space and cerebrospinal fluid. It is most commonly associated with lung and breast cancers and represents a complex and multifaceted disease treated with surgery, radiotherapy, systemic and intrathecal drug treatments. Wang et al. described the state of the art of intrathecal administration of chemotherapeutic, targeted and immunotherapeutic agents for the treatment of LMD, providing safety considerations and recommendations for minimizing adverse effects.

The use of arginine–glycine–aspartic acid (RGD) peptides for tumor targeting represents an innovative and highly selective strategy in cancer therapy. RGD-functionalized nanocarriers enhance drug accumulation and cellular uptake as a consequence of the natural affinity of the RGD motif for integrin receptors, which are frequently overexpressed on tumor cells and their associated endothelial tissue. This targeting can decrease off-target effects on healthy organs. Wang et al. reviewed recent advances deriving from the conjugation of RGD to various nanosystems, such as liposomes, polymeric or inorganic nanoparticles. Nevertheless, potential immunogenicity, peptide degradation, variability of integrin expression among tumors, the need for scalable procedures, and the possibility to exploit this molecule in personalized therapies remain challenges to be addressed.

In the context of expanding peptide-based therapeutic strategies in oncology and following the promising advances observed with RGD-functionalized systems for targeted drug delivery, antimicrobial peptides (AMPs) emerged as a novel and promising class of anticancer agents. Zare-Zardini et al. reported the peculiar properties of AMPs proposed for cancer treatment. In detail, it was shown that their physico-chemical features, such as net charge, length, hydrophobicity and Boman index, influence their ability to destabilize the cancer cell membranes, interfere with intracellular pathways and modulate the tumor microenvironment, suggesting a potential role in drug delivery.

An alternative peptide-independent strategy for targeted drug delivery and cancer therapy is represented by porphyrin-based nanoscale metal-organic frameworks (por-nMOFs) due to their ability in generating cytotoxic reactive oxygen species (ROS) for application in photodynamic therapy (PDT). Unfortunately, por-nMOFs are characterized by a rapid clearance and insufficient tumor targeting. Zou et al. focused on the biomimetic approaches proposed to overcome these limitations, such as the coating por-nMOFs with cell membrane derived from various cells. This stategy allows to modulate the pharmacokinetic profile of por-nMOFs, helps evade the reticuloendothelial system and increases tumor accumulation, demonstrating the potential of these innovative formulations for the development of advanced PDT.

ROS-mediated cytotoxicity was identified as the key mechanism exploited by *Atractylodes lancea* DC, a traditional Asian medicinal herb, as discussed by Ahn et al. In detail, the plant extract demonstrated selective cytotoxicity against prostate cancer cells compared to healthy cells, inducing apoptosis through mitochondrial dysfunction characterized by decreased membrane potential and increased intracellular calcium levels.

Fang et al. investigated the antitumor effects of Schisandrin B, another natural compound obtained by the dried fruits of *Schisandra chinensis.* The study described various molecular targets of Schisandrin B depending on the type of treated tumor, and included a chemoinformatic evaluation of additional intracellular pathways involved in its cytotoxicity. These findings highlight the potential of the active compound as a component of innovative antitumor formulations.

The involvement of CXC chemokine receptor 3 (CXCR3), a G protein-coupled chemokine receptor, in the development of tumor-related diseases was described by Hou et al. Specifically, these authors discussed the design of small molecule antagonists and their *in vitro* and *in vivo* efficacy with the aim of highlighting the potential impact of this approach in the treatment of various human carcinomas.

In summary, the articles of this Research Topic present a range of therapeutic strategies employed or under investigation for the management of cancer. Although studies on several novel molecules or drug delivery systems herein described are still at an early stage of development, the Guest Editors believe that these approaches may hold promise as future pharmacological formulations.
